# Supplementary material for: A Multidrug-Resistant Escherichia coli Caused the Death of the Chinese Soft-Shelled Turtle (Pelodiscus sinensis)
Source: Vet Sci. 2025 May 14;12(5):473. doi: 10.3390/vetsci12050473 (PMC12115518; doi:10.3390/vetsci12050473)
Supplement: Supplementary file 1 [file vetsci-12-00473-s001.zip › vetsci-3563851-supplementary.pdf]

Table S1 Sequences of drug resistance genes primers

Gene	Primer sequence (5'-3′)	Product Size (bp)	Optimal Annealing Temperature (°C)
16SrRNA	F: AGAGTTTGATCATGGCTCAG<br>R: TACGGTTACCTTGTTACGACTT	1500	55
oqxA	F: GATCAGTCAGTGGGATAGTTT<br>R: TACTCGGCGTTAACTGATTA	680	51
oqxB	F: TTCTCCCCCGGCGGGAAGTAC<br>R: CTCGGCCATTTTGGCGCGTA	525	61
aac(3)- II	F: GGCGACTTCACCGTTTCT<br>R: GGACCGATCACCCTACGAG	456	54
blaTEM	F: CCAATGCTTAATCAGTGAGG<br>R: ATGAGTATTCAACATTTCCG	425	57
floR	F: CTGAACACGACGCCCGCTAT<br>R: GGACCGCTCCGCAAACAA	785	60
Mcr4	F: TTGCAGACGCCCATGGAATA<br>R: GCCGCATGAGCTAGTATCGT	207	57
ereA	F: GCCGGTGCTCATGAACTTGAG<br>R: CGACTCTATTCGATCAGAGGC	386	60
aphA1	F: ATGGGCTCGCGATAATGTC<br>R: CTCACCGAGGCAGTTCCAT	700	60

Table S2 Results of biochemical characterization of the bacterial strain HD-593

Reaction item	Result	Reaction item	Result
A1 Negative Control	N	E1 Gelatin	N
A2 Dextrin	B	E2 Glycyl-L-Proline	P
A3 D-Maltose	B	E3 L-Alanine	P
A4 D-Trehalose	N	E4 L-Arginine	N
A5 D-Cellobiose	N	E5 L-Aspartic Acid	B
A6 Gentiobiose	N	E6 L-Glutamic Acid	B
A7 Sucrose	B	E7 L-Histidine	N
A8 D-Turanose	N	E8 L-Pyroglutamic Acid	N
A9 Stachyose	N	E9 L-Serine	P
A10 Positive Control	B	E10 Lincomycin	P
A11 Acidic PH PH6	B	E11 Guanidine HCl	P
A12 Acidic PH PH5	B	E12 Niaproof 4	P
B1 D-Raffinose	B	F1 Pectin	N
B2 α-D-Lactose	N	F2 D-Galacturonic Acid	P

B3 D-Melibiose	B	F3 L L-Galactonic Acid Lactone	P
B4 β-Methyl-D-Glucoside	N	F4 D-Gluconic Acid	P
B5 D-Salicin	N	F5 D-Glucuronic Acid	P
B6 N-Acetyl-D-Glucosamine	B	F6 Glucuronamide	P
B7 N-Acetyl-β-D-Mannosamine	N	F7 Mucic Acid	N
B8 N-Acetyl-D-Galactosamine	B	F8 Quinic Acid	N
B9 N-Acetyl Neuraminic Acid	B	F9 D-Saccharic Acid	P
B10 1% NaCl	P	F10 Vancomycin	P
B11 4% NaCl	B	F11 Tetrazolium Violet	P
B12 8% NaCl	N	F12 Tetrazolium Blue	P
C1 α-D-Glucose	B	G1 P-Hydroxy-Phenylacetic Acid	B
C2 D-Mannose	B	G2 Methyl Pyruvate	B
C3 D-Fructose	B	G3 D-Lactic Acid Methyl Ester	N
C4 D-Galactose	P	G4 L-Lactic Acid	P
C5 3-Methyl Glucose	N	G5 Citric Acid	N
C6 D-Fucose	B	G6 6α-Keto-Glutaric Acid	N
C7 L-Fucose	P	G7 D-Malic Acid	P
C8 L-Rhamnose	P	G8 L-Malic Acid	B
C9 Inosine	P	G9 Bromo-Succinic Acid	B
C10 1%Sodium Lactate	P	G10 Nalidixic Acid	P
C11 Fusidic Acida	P	G11 Lithium Chloride	P
C12 D-Serine	B	G12 Potassium Tellurite	P
D1 D-Sorbitol	B	H1 Tween 40	N
D2 D-Mannitol	B	H2 γ-Amino-Butyric Acid	N
D3 D-Arabitol	N	H3α-Hydroxy-Butyric Acid	B
D4 myo-lnositol	N	H4 β-Hydroxy-D,L-Butyric Acid	N
D5 Glycerol	B	H5 α-Keto-Butyric Acid	N
D6 D-Glucose-6-PO4	P	H6 Acetoacetic Acid	N
D7 D-Fructose-6-PO4	P	H7 Propionic Acid	B
D8 D-Aspartic Acid	B	H8 Acetic Acid	B
D9 D-Serine	N	H9 Formic Acid	B
D10 Troleandomycin	P	H10 Aztreonam	P
D11 Rifamycin SV	P	H11 Sodium Butyrate	P
D12 Minocycline	P	H12 Sodium Bromate	N

Notes: “P”=Postive; “N”=Negtive; “B”=Borderline.

Table S3 Drug susceptibility tests of strain HD-593

Medicine name	content	Inhibition Zone (mm)	Sensitivity
ceftriaxone	30μg	6	R
enrofloxacin	10μg	6	R
doxycycline	30μg	6	R
sulfonamide	300 IU	6	R
gentamycin	10μg	6	R
neomycin	30μg	6	R
florfenicol	30μg	6	R
carbenicillin	100μg	6	R
cefradine	30μg	6	R
erythromycin	15μg	6	R
penicillin	30μg	6	R
ampicillin	10μg	6	R
midecamycin	30μg	6	R
streptomycin	10μg	6	R
furazolidone	100μg	11	I
polymyxin	300 IU	12	I

Notes: S, susceptible; I, intermediate; R, resistant.

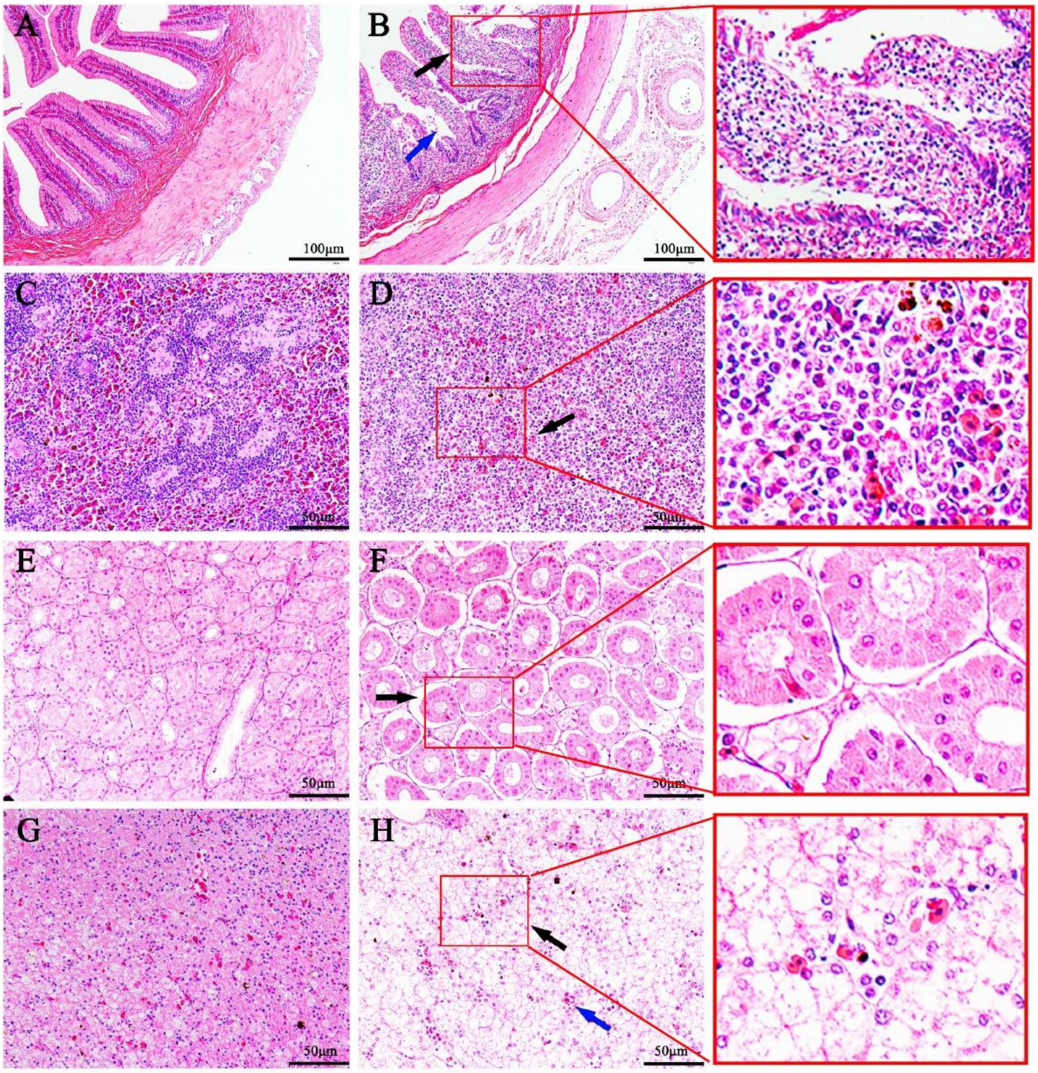

Figure S1. Pathological observations of normal and diseased tissues in *P. sinensis*. A: Intestinal tissue of healthy *P. sinensis*, B: Intestinal villi of sick *P. sinensis* was detached (black arrow), and the epithelial cells of the small intestine were necrotic (blue arrow), C: Spleen of healthy *P. sinensis*, D: Spleen of ill *P. sinensis* with necrotic cells (black arrow), E: Kidney of healthy *P. sinensis*, F: Swelling of glomeruli of diseased *P. sinensis* (black arrow), G: Liver of healthy turtle, H: Liver of sick turtle with severe steatosis (black arrow) and swelling of hepatic sinusoids (blue arrow). Microscopic examination of tissues revealed significant pathological alterations in the diseased P. sinensis. In contrast to the normal tissue architecture observed in healthy P. sinensis, organs such as the liver, kidneys, intestines, and spleen exhibited marked morphological changes in the diseased individuals. A detailed histopathological analysis of the small intestine showed severe damage to the mesenteric villi, including detachment, epithelial cell necrosis, and muscle cell necrosis. Additionally, the external mucous membrane displayed pronounced swelling (Figure S1B). The spleen of diseased P. sinensis displayed widespread necrosis, with loss of the clear demarcation between red and white pulp. Splenic cells were necrotic and displayed marginated nuclear material (Figure S1D). Examination of the kidneys revealed glomerular swelling, epithelial cell necrosis, and inflammatory cell infiltration within the renal tubules (Figure S1F). The livers of diseased P. sinensis exhibited severe steatosis, characterized by marginated nuclear material, distended hepatic sinusoids, and infiltration of inflammatory cells (Figure S1H).

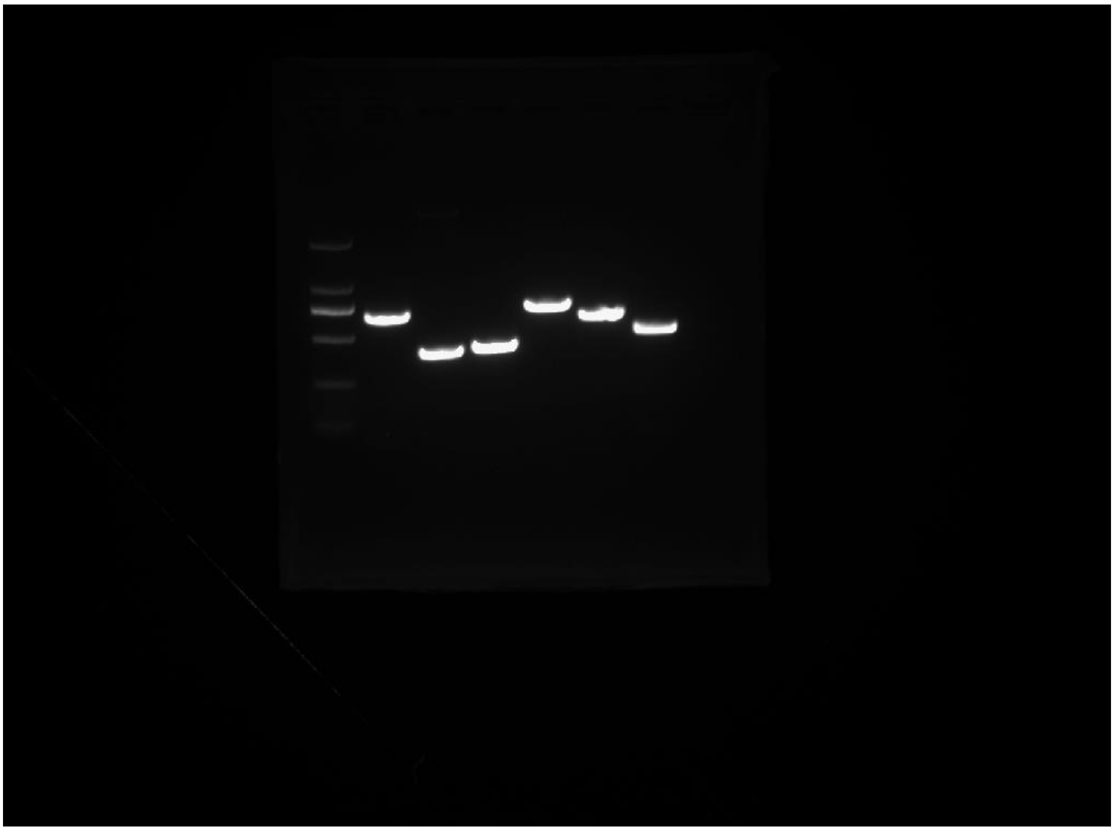

Figure S2. Original gel image.
